# Development of a self-management mobile app for bereaved adolescents: evaluating patient and public involvement

**DOI:** 10.1080/20008066.2024.2375139

**Published:** 2024-07-12

**Authors:** Rebecca Rhodin, Rakel Eklund, Anneli Silvén Hagström, Atle Dyregrov, Josefin Sveen

**Affiliations:** a Department of Women’s and Children’s Health, Uppsala University, Uppsala, Sweden; b Department of Social Work, Stockholm University, Stockholm, Sweden; c Center for Crisis Psychology, University of Bergen, Bergen, Norway

**Keywords:** Bereavement, ehealth, evaluation, grief, intervention development, loss, parentally bereaved, PPI, psychosocial support, teenagers, Duelo, esalud, evaluación, duelo, desarrollo de intervenciones, pérdida, duelo parental, PPI, apoyo psicosocial, adolescentes

## Abstract

**Background:** Losing a family member during childhood is a potentially traumatic event and increases the risk of mental health difficulties. Adolescents have the right to express their views in research of relevance to them, but few studies have involved bereaved adolescents as collaborators (i.e. Patient and Public Involvement (PPI)). Furthermore, to ensure meaningful and non-tokenistic involvement, bereaved adolescents’ levels of participation and experiences of taking part in research need to be evaluated.

**Objectives:** The aim was to describe and evaluate a PPI process working with bereaved adolescents to develop a self-management mobile app for adolescents in grief.

**Methods:** The PPI process consisted of four workshops during which the app’s logo, colours, name, content, and layout were discussed with six parentally bereaved adolescents aged 13–18 years. The adolescents were recruited through a non-profit organisation providing support for adolescents in grief. The PPI process was documented and evaluated using participant observations and an online survey completed by the adolescents, covering the themes of social context, participation, and influence.

**Results:** The adolescents perceived the social context as comfortable and inclusive, where their knowledge was valued. Their participation was characterised by ownership and motivated by a desire to help others with similar experiences. The adolescents’ ability to participate in PPI activities was assisted by the researchers’ flexibility, although challenging assignments may have made participation harder. Throughout PPI activities, adolescents contributed with relevant input and reported feeling influential. The study reached the intended levels of participation and appeared to adequately fulfil the adolescents’ right to participation.

**Conclusions:** Engaging adolescents who have undergone a potentially traumatic event, such as the loss of a family member, in research can enhance the overall relevance of the study. Moreover, it can entail a meaningful and positive experience for the participating adolescents, while also fulfilling their fundamental right to participation.

## HIGHLIGHTS

- A collaborative process with parentally bereaved adolescents to develop a psychosocial self-management mobile app for adolescents in grief was described and evaluated.
- Adolescents made significant contributions, took ownership, and experienced having influence over the decisions made, which increased the relevance of the intervention.
- The collaborative process reached the intended level of participation and created a positive and meaningful experience for the adolescents.

## Abstract (es)

**Antecedentes:** Perder a un miembro de la familia durante la niñez es un evento potencialmente traumático y aumenta el riesgo de dificultades de salud mental. Los adolescentes tienen derecho a expresar sus puntos de vista en investigaciones relevantes para ellos, pero pocos estudios han involucrado a adolescentes en duelo como colaboradores (es decir, Participación Pública y del Paciente (PPI, por sus siglas en inglés)). Además, para garantizar una participación significativa y no simbólica, es necesario evaluar los niveles de participación y las experiencias de participación en la investigación de los adolescentes en duelo.

**Objetivos:** El objetivo fue describir y evaluar un proceso de PPI que trabaja con adolescentes en duelo para desarrollar una aplicación móvil de autogestión para adolescentes en duelo.

**Métodos:** El proceso de PPI consistió en cuatro talleres durante los cuales se discutió el logotipo, los colores, el nombre, el contenido y el diseño de la aplicación con seis adolescentes de entre 13 y 18 años, en duelo por alguno de sus padres. Los adolescentes fueron reclutados a través de una organización sin fines de lucro que brinda apoyo a adolescentes en duelo. El proceso de PPI fue documentado y evaluado mediante observaciones participantes y una encuesta en línea completada por los adolescentes, que cubre los temas de contexto social, participación e influencia.

**Resultados:** Los adolescentes percibieron el contexto social como cómodo e inclusivo, donde se valoraban sus conocimientos. Su participación se caracterizó por la apropiación y fue motivada por el deseo de ayudar a otros con experiencias similares. La capacidad de los adolescentes para participar en las actividades de PPI se vio favorecida por la flexibilidad de los investigadores, aunque las tareas desafiantes pueden haber dificultado la participación. A lo largo de las actividades de PPI, los adolescentes contribuyeron con aportes relevantes y reportaron sentirse influyentes. El estudio alcanzó los niveles de participación previstos y pareció cumplir adecuadamente el derecho de participación de los adolescentes.

**Conclusiones:** Involucrar en la investigación a adolescentes que han pasado por un evento potencialmente traumático, como la pérdida de un miembro de la familia, puede mejorar la relevancia general del estudio. Además, puede suponer una experiencia significativa y positiva para los adolescentes participantes, cumpliendo al mismo tiempo con su derecho fundamental a la participación.

## 1. Introduction

Losing a family member is potentially one of the most traumatic events an adolescent can experience, increasing the risk of mental health difficulties (Lytje & Dyregrov, 2019). Early interventions can be helpful for adolescents after traumatic events (Kramer & Landolt, 2011) and eHealth interventions are showing promise in supporting trauma-exposed persons (Bakker et al., 2020). There is, however, a lack of evaluated psychosocial interventions to support adolescents in grief and more research concerning the means to guide these young mourners in navigating the complexities of grief is therefore required (Bergman & Hanson, 2014). Bereaved adolescents also need to be involved in the conceptualisation and formulation of interventions, particularly given the existing knowledge gap regarding what elements that adolescents perceive as supportive in their grieving process (Bergman & Hanson, 2014).

Patient and Public Involvement (PPI) is defined as research carried out ‘with or by’ members of the community rather than ‘to or for’ them (INVOLVE, 2012) and entails involving public contributors with relevant experience. The rationale for using PPI ranges from the ethical argument that the public has a right to be actively engaged in research funded by the public to empirical evidence of how PPI contributes to research quality and the relevance of interventions (Brett et al., 2014; Gradinger et al., 2015). Inconsistent reporting on PPI endeavours and a lack of rigorous evaluation tools has, however, inhibited the improvement in quality of PPI undertakings (Boivin et al., 2018; Staniszewska et al., 2017). Among prior studies involving young people in developing digital mental health interventions, most of which use cognitive behavioural therapy to address symptoms of depression or anxiety, few have evaluated the PPI process (Bevan Jones et al., 2020). Notably, the predominant focus of evaluations on PPI’s impact on research outcomes has also diverted attention away from how PPI is practised and its potential adverse effects. There is, therefore, a need to investigate how public contributors are affected by their participatory roles (Russell et al., 2020).

While reporting on PPI initiatives with adolescents is lacking, indications suggest that adolescents can derive benefits from participation, such as gaining valuable experiences, skills, feelings of enjoyment and appreciation; feeling listened to; and being empowered to affect positive change. However, the levels of involvement reached in PPI endeavours with adolescents have varied significantly (Larsson et al., 2018; Rouncefield-Swales et al., 2021 Dec) and a perception that adolescents lack the requisite experience to be influential in research may contribute to creating hierarchical PPI settings (Dovey-Pearce et al., 2019). There are, therefore, risks of unintended consequences relating to power dynamics and tokenistic involvement in adolescent-focused PPI efforts (Rouncefield-Swales et al., 2021). Andriessen et al. (2022) underscore concerns raised by research ethical committees regarding the potential of causing harm to bereaved adolescents when including them in research. However, studies have indicated that both bereaved adolescents and adults, including young adults parentally bereaved during childhood, consider participation in research to be a meaningful opportunity to share their experiences (Andriessen et al., 2022; Smith et al., 2018; Udo et al., 2019).

Participatory research involving adolescents with potentially traumatic experiences includes, for example, one study of refugee youth participating in intervention adaptation (Pérez-Aronsson et al., 2022) and others using participatory art methods with youth who have experienced adverse childhood events (Pavarini et al., 2021). Researchers working with potentially traumatised adolescents through participatory methods emphasise the importance of power-sharing, of allowing the adolescents to lead with researcher support, clarifying roles and responsibilities from the onset, and maintaining open communication with the adolescents between meetings, without pressuring them into sharing personal experiences and risk re-traumatisation (Pavarini et al., 2021; Sarkadi et al., 2023). Despite these considerations, studies involving adolescents with experiences of potentially traumatic events, such as bereavement, in PPI remain scarce.

Not involving bereaved adolescents as public contributors in research can be argued to reflect a failure to accommodate their rights, as articulated in Article 12 of the United Nations Convention on the Rights of the Child (UNCRC), which emphasises the right of children and adolescents to participate in decision-making processes that affect them. However, fulfilling the principles of the UNCRC requires a level of involvement where adolescents’ views are, at a minimum, taken into account (Shier, 2001). There is, therefore, a need to assess the levels of involvement achieved when integrating bereaved adolescents into PPI initiatives. Shier’s framework (Shier, 2019) proposes a model for planning and evaluating research with children and adolescents that delineates four levels of involvement: non-involvement, consultation, collaboration, and decision-making. This framework contends that higher levels of involvement are not always preferable, since this does not necessarily make participation more inclusive or empowering for the adolescents nor does it automatically strengthen the research. Instead, the need for researchers to be purposeful and flexible, in order to engage adolescents appropriately in meaningful ways, is highlighted (Shier, 2019).

In summary, to address the gap in understanding concerning the support needs of bereaved adolescents, their inclusion in research and the development of supportive interventions is crucial. This involvement should align with the UNCRC, recognising bereaved adolescents’ right to participation. However, to ensure meaningful and non-tokenistic involvement, in line with the UNCRC principles, an evaluation of bereaved adolescents’ experiences of participating in the PPI process becomes essential. Therefore, this study aimed to describe and evaluate the PPI process of working with bereaved adolescents as public contributors in developing a self-management mobile app tailored for adolescents in grief.

## 2. Method

### 2.1. Setting

The study was conducted in collaboration with a non-profit organisation (NPO) that provides support for grieving children in a large Swedish city. PPI activities for this study took place between November 2022 and April 2023. The study aimed to develop a psychosocial self-management mobile app for adolescents experiencing grief due to the loss of a parent and/or sibling. The app will be evaluated through an upcoming randomised controlled trial (Clinicaltrials.gov, identifier: NCT06093113) during which PPI activities with the adolescents will continue.

Adolescent involvement levels in PPI were predefined using Shier’s framework (Shier, 2019). The plan encompassed collaborative development of the research instrument, i.e. the app, and consultation with adolescents during data collection through surveying their opinions. Adolescents were, therefore, not intended to make decisions regarding the app or the study by themselves, but to collaborate with researchers. This delineation was consistently communicated to the adolescents.

The four researchers who designed the project (JS, RE, ASH and AD) brought different perspectives into the study, with backgrounds in nursing, social work and psychology including clinical experience working with adolescents. Researcher RR, a PhD student with a background in global health, did not participate in the planning of the project and intended involvement levels; instead, RR brought a new perspective into the study when striving to understand the adolescents’ experiences of the PPI process.

### 2.2. Adolescents as public contributors

Eligible public contributors were adolescents aged 12–19 years who had experienced the loss of a parent and/or sibling and had access to a smartphone, the latter being required to ensure adolescents’ ability to take part in app testing. The NPO helped recruit those who had received support from their organisation, which allowed the NPO staff to assess whether the adolescents were emotionally ready to participate. The adolescents and their guardians were approached by NPO staff and provided with verbal and written information about the study. Forms were then sent by researchers to the adolescents via mail, as well as to the guardians of those under 18 years old, for them to provide written informed consent.

Seven adolescents initially consented to participate; however, one did not attend any of the PPI activities. The group comprised four girls and two boys, aged 13–18 years, native Swedish speakers, residing in the same large Swedish city and having experienced parental loss due to various causes, including for example disease and suicide. Notably, some adolescents were familiar with each other from prior interactions within the same support group.

### 2.3. PPI activities

The PPI activities comprised four 2-hour workshops held at the NPO and attended by the adolescents, two members of the research team, and a NPO staff member already known to the adolescents. The workshops were planned and led by researcher RE. The adolescents’ age and grief experiences were taken into account during PPI planning and included measures such as scheduling breaks and sending reminders before each workshop. Whereas the format of the initial workshop was determined by the researchers, the format, content, and setting of subsequent meetings were collaboratively decided with the adolescents. All workshops were held on weekday evenings after school and included dinner. Pre-prepared agendas for the workshops were occasionally amended according to the adolescents’ input and needs. The workshops involved group discussions and brainstorming sessions, utilising creative tools such as post-it notes and tablets, where the app features were discussed as outlined in Table 1. Adolescents received a multi-store gift card of 250 SEK as compensation for attending each workshop.

**Table 1.** Attendees and content of PPI workshops

	Workshop 1	Workshop 2	Workshop 3	Workshop 4
Attendees	Researcher RE*, researcher RR, NPO staff, five adolescents	Researcher RE*, researcher RR, four adolescents	Researcher RR*, researcher JS, NPO staff, five adolescents	Researcher RE*, researcher RR, NPO staff, two adolescents
Content	Introduction to the project and to each other	Introduction, recap of the previous workshop, and discussions about the progress made	Introduction, recap of the previous workshops, and discussions about the progress made	Introduction, recap of the previous workshops, and discussions about the progress made
	Brainstorming app logo	Brainstorming overall app ideas	Feedback on revised content and structure	Brainstorming the type of avatar
	Discussing colours	Brainstorming app content	Naming app sections	Brainstorming symbols and icons
	Brainstorming app names	Structuring app content into sections	Discussing measuring grief and emotions	Evaluation of PPI activities

Note: * Researcher leading the workshop.

Between the workshops, the research team processed the material produced in collaboration with the adolescents and consulted members of an advisory group for input. This group comprised a young adult with bereavement experience, a grief psychotherapist/researcher, an NPO staff member, and a user experience (UX) designer. Concurrently, the adolescents received project updates via text messages from the researchers and were invited to provide feedback on new developments.

Attendance at the workshops varied (see Table 1). Before workshop 3, the leading researcher (RE) became ill so researcher JS stepped in and researcher RR, who normally documented the workshops, took a more leading role. Regrettably, these adjustments were not communicated to the adolescents before the workshop.

### 2.4. Data collection

Sociodemographic data on the adolescents, including age, gender, and home address, were acquired from the NPO via telephone at the beginning of the study. Throughout the workshops, participant observations were conducted to document the PPI process from a researcher’s perspective. While RE facilitated the workshops, RR documented social interactions, and processes taking place by collecting detailed fieldnotes of, for example, activities, actions, goals and affections, while also taking part in group discussions (Reeves et al., 2008). The adolescents were aware of these field notes being taken and why. The observations adhered to an observation schedule (see supplement Table 1) inspired by the ‘Active Involvement of Users in Research Observation Schedule’ (Warner et al., 2021). This schedule focused on three key themes: social context (e.g. are there positive and negative interactions?), participation (e.g. are contributors participating actively or passively?) and influence (e.g. are contributors influential in decision-making?). After the workshops, the field notes were elaborated upon and clarified. They were then checked by the other participating researcher after which revisions and supplements were made before being shared with others in the research group. The final observation notes consisted of 16 pages. Additionally, adolescents’ suggestions, often in the form of brainstormed post-it notes, were collected and compiled to document the workshops.

Furthermore, adolescents were invited to share their experiences of participating in the project via an online survey, developed from the ‘Research Engagement Survey Tool’ (Goodman et al., 2017) and the ‘Active Involvement of Users in Research Questionnaire’ (Warner et al., 2021). Aligned with the observation schedule’s themes, the survey featured 14 questions with responses rated on a 5-item Likert scale from ‘always’ to ‘never’ and four open-ended questions. Survey data collection occurred at the end of the final workshop through the REDCap electronic data management tool. Adolescents not present at the final workshop received text messages prompting survey completion, with some being sent several reminders, upon which all six adolescents completed the survey.

### 2.5. Data analysis

A mixed methods approach with a convergent design was employed to attain a comprehensive understanding of how the PPI process was experienced. This entailed observations, quantitative survey data, and quotes from the open-ended survey questions being combined and compared post-analysis (Creswell & Plano Clark, 2018). The GRIPP2 international guidelines (Staniszewska et al., 2017) and checklist for reporting on PPI were used to guide the content of the article.

Thematic analysis (Braun & Clarke, 2006) was applied to analyse the observation notes, aiming to discern and interpret patterns within the data. The analysis is derived from a social constructionist epistemology where themes and reality are viewed as socially produced; the analytical focus centres on understanding how the social context enables the observed behaviours. Themes of relevance to the study’s aim, initially defined during the observation schedule construction, were consistently maintained throughout the analysis. After the first step of familiarisation with the observation notes, initial codes (e.g. ‘jokes and shared laughter’ and ‘changing roles’) related to the themes of the observation schedule (social context, participation and influence) were generated. Codes were then grouped and preliminary subthemes were inductively produced, described and interpreted by researchers RR and JS in collaboration with the research group, comparing interpretations and providing feedback. Subsequently, reviews and revisions were made to ensure alignment with the dataset and increase the nuance and richness of subthemes. Themes and subthemes were then named, defined, and adjusted collaboratively with the research team until consensus was reached (Braun & Clarke, 2006). Descriptive statistics were employed for the analysis of quantitative survey data.

### 2.6. Ethical considerations

The study received approval from the Swedish Ethical Review Authority (project no. 2022-04101-01). The evaluation of PPI activities entailed the adolescents being public contributors and research participants, since data were collected from them through participant observations and survey responses. Ethically, this distinction is crucial since clarity concerning the different roles is central to the adolescents’ informed consent. Clarifications were provided continually regarding which roles applied to which activities to ensure their awareness and consent. Consistent with Martineau’s recommendations (Martineau et al., 2020) for mitigating ethical issues in PPI, informed consent was approached as a continuous process rather than a singular event.

Another pertinent ethical concern in PPI involves the risk of causing harm to public contributors (Martineau et al., 2020). Given that bereaved adolescents constitute a group with potentially traumatic experiences, efforts were made to minimise distress during PPI activities. This included not requiring adolescents to discuss their own loss experiences but creating space for those choosing to do so. Support was offered throughout the project by the NPO.

## 3. Results

The results are presented below according to the themes and subthemes outlined in Table 2. The observation results are mixed and compared with adolescents’ quantitative survey responses (Table 3).

**Table 2.** Overview of themes and subthemes.

Theme	Subtheme	Description
A social context beneficial to participation	A safe space for sharing grief and experience joy and inclusion	Indications of a comfortable and encouraging group setting inclusive of different voices and opinions, along with contributing factors
Ensuring meaningful participation by recognising adolescent needs	Project ownership facilitated by information and recognition	Indications of adolescents’ participation being characterised by a feeling of ownership and potential contributing factors
	Flexibility to navigate changing needs and encountered challenges	Indications of how flexibility was used to manage potential barriers and facilitate participation
Influence shaped by adolescent contributions and study logistics	Insightful contributions enhancing adolescents’ influence	Indications of adolescents’ contributions being characterised by relevance and perceptiveness, increasing their impact
	Varying opportunities for influence and decision-making	Indications of adolescents attaining various ways and opportunities for influence, but not being involved in final decision-making

**Table 3.** Adolescents’ survey responses (*n* = 6).

Researchers and NPO staff have …	Always	Often	Some-times	Rarely	Never	Not applicable
1. Shown appreciation for the time I have put into the work	5	1				
2. Kept me informed about what was happening in the project	5	1				
3. Helped me with personal problems	1	2	2			1
4. Used my ideas in the project	3	2	1			
5. Changed the format if I made new suggestions	3	1	2			
6. Encouraged us to learn from each other	4	1	1			
7. Handled situations within the group fairly if we did not get along	3	1				2
8. Shown that my ideas are equally as important as theirs	5	1				
9. Enabled everyone’s voices to be heard	6					
10. Made final decisions which mirrored ideas from everyone in the group	4	2				
11. Treated me with openness and respect	6					
12. Made me feel that my opinions are valuable	6					
13. Made it hard for me to be involved because the language and/or assignments have been difficult to understand			1	1	4	
14. Disregarded or ignored my suggestions				1	5	

### 3.1. A social context beneficial to participation

This theme explores characteristics of the social context. The social context is recognised as an essential requirement for meaningful participation, since a conducive context can empower and facilitate adolescents to participate in ways that are meaningful for them and the project. The subtheme ‘A safe space for sharing grief and experience joy and inclusion’ was identified.

#### 3.1.1. A safe space for sharing grief and experience joy and inclusion

During the workshops, jokes and shared laughter were observed as common occurrences within the group. The adolescents willingly disclosed their personal loss experiences and struggles, and made numerous expressions of appreciation for the group. For example, the adolescents expressed how much fun it had been to participate in the group, and that they could not wait for future sessions. Notably, one adolescent expressed how he had experienced a fear of working with researchers but then described the experience as positive. It was observed that the adolescents appeared familiar with and comfortable in the workshop space, likely due to their previous experiences of visiting the NPO while, the researchers were the visiting parties. The adolescents’ ease in navigating the workshop setting, including retrieving needed tools, suggested that this familiarity contributed to the positive and safe group setting. Additionally, observations revealed consistent efforts from both adolescents and researchers to ensure that everyone was invited to speak, as well as conflicting suggestions being encouraged and respectfully discussed. For example, during the first workshop, an adolescent hesitated to make a suggestion, saying it was probably stupid, but then received reassurance and appreciation from the other adolescents.

The survey responses were supportive of the social context being perceived as positive, with all adolescents stating that they always felt treated with openness and respect. Additionally, the majority also stated that they had been helped with personal problems. One adolescent (no. 5), responding to how the social context was experienced wrote: ‘We have been close to each other and had a lot of fun’. Survey responses also aligned with observations of inclusiveness, with all adolescents stating that researchers and NPO staff enabled everyone’s voices to be heard. Additionally, the majority stated that disagreements within the group were handled fairly by the researchers. One adolescent (no. 2) wrote: ‘Working together and making decisions to make the app as awesome as possible has worked really well. Everyone has been allowed to voice their ideas and opinions about the app’.

### 3.2. Ensuring meaningful participation by recognising adolescent needs

This theme explores adolescent participation during the workshops and factors that may have influenced their ability to take part in a meaningful way. Participation is considered crucial for the adolescents to exert influence in the research project. Identified subthemes include: ‘Project ownership facilitated by information and recognition’ and ‘Flexibility to navigate changing needs and encountered challenges’.

#### 3.2.1. Project ownership facilitated by information and recognition

Throughout the workshops, the adolescents were observed to become increasingly invested in the project, taking on additional tasks beyond those required, and expressing their appreciation and desire to remain involved in upcoming phases of the research. It was also observed that the adolescents were approached as being the experts in the workshop setting, which may have contributed to the displayed engagement with the project. For example, when the adolescents discovered that, unlike them, the adult advisory group did not receive financial compensation, their initial surprise turned into understanding, with one adolescent acknowledging that the adolescents were the experts. In addition, the provision of easily accessible information was essential to empower adolescents to assume more leading roles and promote project ownership. This included contextualising tasks, and offering updates promoting a comprehensive project understanding beyond workshop topics.

The researchers appear to have been successful in providing information, as the majority of the adolescents stated in the survey that they were consistently informed about project development. The adolescents also appear to have experienced being treated as having valuable knowledge within the workshop setting since, in the survey, the majority stated that they felt that mutual learning was encouraged by the researchers and NPO staff. All the adolescents also stated that they always felt appreciated during the workshops and that their opinions were consistently valued. However, adolescents’ commitment also appeared related to a desire to help others with similar experiences, which was evident in the majority of the adolescents’ responses to the open-ended question concerning whether participation had been perceived as meaningful. For instance, one adolescent (no. 3) wrote: ‘Most of all it has been interesting and educational. It feels good to participate in something that can actually help people’ and another adolescent (no. 4) similarly wrote: ‘Makes me happy to think that I can help others and make a difference’. These altruistic motivations, and the belief in their capacity to make a difference through the project, may, along with information and recognition, have contributed to the observed level of ownership.

#### 3.2.2. Flexibility to navigate changing needs and encountered challenges

The observations indicated that tiredness impacted adolescents’ participation, hindering their ability to contribute. Signs such as yawning, difficulty keeping to the discussions, reduced number of suggestions, and decreased group interaction were evident when tiredness set in. On such occasions, the researchers needed to assume more leading roles to help maintain focus and creativity. Another potential barrier pertained to the nature of the assignments and the workshop set-up. While most brainstorming sessions were lively and productive, some tasks were observed as posing challenges for the adolescents. This arose when tasks were perceived as too challenging or abstract and demanding considerable creativity. Conversely, overly concrete assignments appeared to inhibit creativity and restrict the adolescents’ freedom of thought. This was visible, for example, during the third workshop when adolescents were tasked with providing feedback on app content previously brainstormed. In contrast to brainstorming sessions, this task resulted in less fruitful discussions during which the adolescents’ abilities to contribute and participate in a meaningful way appeared limited.

Various factors held importance for managing these potential barriers to adolescents’ participation, including their autonomy in deciding how to participate and contribute to the workshops. This entailed choosing whether to work more individually or collaboratively, more vocally or internally, stepping away when needed, and being able to use tools such as tablets, smartphones, fidget spinners, and post-it notes to foster creativity and maintain focus. This flexibility was particularly important given the changing capacities of the adolescents during and between workshops. While they led discussions in some parts, they required additional guidance in more challenging sections or toward the workshop’s conclusion. The researchers’ adaptability, allowing shifts in roles and adjusting workshop set-ups as new insights emerged, was pivotal.

Despite potential barriers, the adolescents do not appear to have experienced difficulty participating, since the majority stated in the survey that researchers and NPO staff never made it difficult for them to participate by using language and/or assignments that were hard to understand. Acknowledging tiredness and varying capacities without seemingly regarding it as a barrier, one adolescent (no.3) wrote: ‘People have been tired and have had worse days, but everyone has been nice and has treated each other respectfully. I think there has been a good and non-rigid atmosphere throughout the meetings and there were no arguments or anything’.

### 3.3. Influence shaped by adolescent contributions and study logistics

This theme pertains to the opportunities available for the adolescents to influence decisions within the project and factors that may have shaped that influence, including characteristics of their contributions. Identified subthemes were: ‘Insightful contributions enhancing adolescents influence’ and ‘Varying opportunities for influence and decision-making’.

#### 3.3.1. Insightful contributions enhancing adolescents’ influence

The adolescents’ contributions were repeatedly observed to be relevant to the topic, meaning they were pertinent from a researcher's perspective. They were additionally well-supported and, for example, articulated the benefits of specific app features for the target population. The adolescents were also observed to identify and problematise novel areas of importance for the app. For instance, they raised concerns about how to avoid the app being exclusive of adolescents who had lost a sibling rather than a parent, and demonstrated awareness of how certain suggestions could be impracticable due to budget constraints. The adolescents also addressed the challenge of promoting app usage since it dealt with a difficult topic and, of their own volition, discussed strategies to make the app supportive rather than melancholy in order to ensure its sustained use by other adolescents. The relevance and perceptiveness of the adolescents’ contributions underscored the significance of their ideas to the app’s development.

That the adolescents’ contributions were found relevant and valued appears to have been conveyed to the adolescents, since all of them stated in the survey that they felt that their ideas were of equal importance to those of the researchers. In response to whether they felt they had been able to influence the app development process, one adolescent (no. 3) wrote: ‘Yes, it feels like everyone listens to each other and builds on each other’s ideas. We haven’t always agreed with one another, but have anyway always been able to get on. I feel that my ideas have been given space in the project’.

#### 3.3.2. Varying opportunities for influence and decision-making

The adolescents were observed as being continuously invited to be involved in shaping the workshop format and actively contributing to decisions, such as setting parameters for giving feedback within the group and modifying the workshop agenda. While many aspects of the workshop format remained unchanged, according to the adolescents’ preferences, their opinions were consistently sought, providing opportunities for them to impact the PPI activities. Additionally, collected post-it notes containing adolescent suggestions provided the basis for decisions regarding the app and the overall study. However, observations revealed that these decisions rarely occurred during the workshops. Input from other members of the research team and the app developer was often necessary to assess the feasibility of suggestions, leading to final decisions being made outside of PPI activities. Although the adolescents’ contributions formed the foundations for these decisions, they were not actively involved in the decision-making discussions. In addition, as the app features became more concretised and what was needed from the adolescents became more specific, instances where certain features were presented as already established were increasingly observed, diminishing the adolescents’ ability to influence them throughout the PPI process.

The adolescents, in line with the observations, appear to have felt influential over PPI activities, with the majority stating in the survey that their ideas were ‘always’ or ‘often’ incorporated into the project and that they felt that the workshop format was changed when new suggestions were made. Despite the adolescents not being present during most final decisions, they also appear to have felt influential in the project overall. In the survey, the majority stated that the researchers and NPO staff never disregarded or ignored their suggestions, and always made final decisions mirroring ideas from everyone in the group. One adolescent (no. 2) wrote: ‘Everyone has been really involved and it really feels like they have taken into account what we feel and our ideas, and that they appreciate our ideas a lot’.

## 4. Discussion

The study has described and evaluated a PPI process that involved bereaved adolescents in the development of a self-management mobile app. The results suggest that the PPI process has worked well, representing a positive experience for both adolescents and the research project. This positive outcome is attributed to various factors, including the NPO recruitment of adolescents and the hosting of workshops at their facilities. This provided a familiar setting for the adolescents and positioned researchers as visitors, potentially mitigating inherent power imbalances since the adolescents had local knowledge which the researchers lacked. Additionally, the adolescents’ pre-existing group interactions, and thereby familiarity with each other, allowed a comfortable social context to be quickly established. Cultivating a positive social context may, therefore, require additional time when working with groups where members are less familiar with each other and the setting.

As demonstrated throughout the PPI process, and emphasised by Shier (2019), flexibility is crucial when engaging adolescents. Unexpected challenges, such as the sudden illness of the workshop leader, underscored the importance of adaptability. Conducting the workshop as planned, with another researcher stepping in, allowed it to move forward and was beneficial for the PPI process, but also revealed insights into how participant changes may impact the social context. More limited discussions during this workshop highlighted the significance of the leading researcher’s role and the importance of informing adolescents in advance of such changes. This workshop also employed a less creative format, emphasising that assignments focused on feedback rather than brainstorming may be less engaging and impede participation. Flexibility was also required when addressing barriers encountered, such as tiredness. The adolescents’ age and grief were considered during the project's design phase, and adjustments were made to keep workshops short, interactive, and creative, and to incorporate breaks and food. Equally important, however, was the flexibility of the researchers in allowing adolescents to participate in a way that suited them, acknowledging their tiredness and providing additional support when necessary to enable meaningful participation.

Despite the challenges encountered and the lessons learned, the researchers’ perception that the PPI process worked well and was a positive experience appears to be shared by the adolescents, who expressed appreciation for being part of the project. This seems to be linked to the opportunity to help other bereaved adolescents with similar experiences, aligning with previous research highlighting how adolescents, through PPI, can feel empowered to create positive change (Rouncefield-Swales et al., 2021). The adolescents also noted that participating in the research was both fun and educational, resonating with previously reported benefits of PPI with adolescents, where involvement is seen as enjoyable and provides contributors with valuable skills (Rouncefield-Swales et al., 2021). The study results are consistent with prior participatory research involving adolescents with potentially traumatic experiences (Pavarini et al., 2021; Sarkadi et al., 2023), emphasising the importance of clear communication about roles and responsibilities, and staying connected between meetings. Our reflections also align with previous research highlighting the significance of being mindful of not pressuring adolescents to share personal stories and taking measures to manage power imbalances, crucial aspects in generating a positive experience for the adolescents.

### 4.1. Levels of participation

While this study did not specifically evaluate the adolescents’ impact on the research, their significant contributions to the project were evident, introducing app features that would not have been envisaged without their input. Although adolescents have an inherent right to be included in research and should not have to prove their worth, their valuable contributions highlight the importance of researchers challenging assumptions about adolescents’ lack of capabilities, as emphasised by Dovey-Pearce et al. (2019). Despite their instrumental role in developing the app and shaping PPI activities, adolescent collaborators did not participate in decision-making discussions as these occurred outside of workshops. While this aligned with the intended levels of participation and was understood by the adolescents, additional consideration during the planning stage could have enabled adolescents’ involvement in these discussions. Considering and evaluating such factors, as highlighted by Shier (2019), enhances researchers’ awareness and intentionality when partnering with adolescents and mitigates the risk of involvement becoming tokenistic (Rouncefield-Swales et al., 2021).

Although not involved in final decisions, the adolescents reported feeling heard and valued, aligning with previously reported benefits of PPI where participation fosters a sense of being listened to (Rouncefield-Swales et al., 2021). More influence or responsibility may hence not always be beneficial, as decision-making can be demanding without necessarily enhancing adolescents’ sense of influence. This aligns with Shier’s (2019) framework, suggesting that meaningful participation does not always require higher involvement levels, although it is important to reflect upon the levels intended and the levels reached. Reviewing the PPI process through this framework, it operated predominantly at the level of collaboration, where adolescents and researchers performed tasks jointly, and occasionally at the consultation and decision-making levels. All these three levels signify that the adolescents’ views have been taken into account, which suggests that the PPI process has adequately aligned with the principles of the UNCRC. Additionally, differing levels of involvement likely contributed to making participation meaningful for both adolescents and the project, by ensuring appropriate and enjoyable forms of engagement and allowing sensitivity to the adolescents’ varying needs and capabilities.

### 4.2. Lessons learned

The PPI process presented several learning opportunities which can help guide future PPI endeavours involving adolescents. First, we would advise putting effort into producing a suitable environment, taking measures to ensure public contributors’ comfort and striving to make activities stimulating for the adolescents. Equally important is the flexibility to adapt PPI assignments and setups when challenges arise. Secondly, approaching adolescents as experts and valuing their contributions is key for making participation meaningful and fostering project engagement. However, enabling adolescents to assume more active roles beyond solely being consulted also requires researchers’ support, being flexible in one’s role, and the provision of comprehensive information to ensure a broad understanding of the project beyond specific PPI activities. Finally, intentionality in planning the levels of involvement and clearly communicating the opportunities for influence available to the adolescents is advisable.

### 4.3. Strengths and limitations

Recruiting adolescents from the support groups of an NPO had ethical benefits through enabling staff to assess adolescents’ emotional readiness for participating in the research. However, this also means that these adolescents had received more help in processing grief and potential trauma than many other bereaved adolescents. All were Swedish-born and lived in the same large Swedish city, raising concerns about result transferability and the applicability of PPI contributions to bereaved adolescents from other backgrounds. Participation in PPI, while experienced positively in this study, could be perceived differently by adolescents who have not received similar support. The inherent power imbalance in the researcher-adolescent relationship, and how adolescents were financially compensated, are also important considerations. These factors may impact how comfortable adolescents feel in responding honestly to the survey, particularly given the small respondent number potentially raising anonymity concerns. In addition, conducting observations while actively engaging in the social context inevitably influences the setting, posing a methodological challenge in ensuring that all relevant details are noted. This particularly applied to the third workshop, where the researcher conducting participant observations also assumed a more leading role. The results may have been different if the researcher had solely observed without participating. However, employing a mixed methods approach enables a comprehensive understanding of how the PPI process was experienced by allowing direct comparison between adolescents’ perspectives and researchers’ standpoints (Creswell & Plano Clark, 2018).

## 5. Conclusions

In summary, the PPI process fostered a positive social context where parentally bereaved adolescents made significant contributions, took ownership, and influenced decisions. The results suggest that the intended levels of participation were achieved and that the PPI endeavour adequately fulfilled the UNCRC principles for participating adolescents. Consequently, our results support the notion that the involvement of potentially traumatised adolescents in research and intervention development enhances intervention relevance and can entail a positive experience for the adolescents while also fulfilling their rights. Given these indications that PPI can be conducted safely under suitable conditions, ethical concerns generating hesitancy in promoting research involving groups who have had potentially traumatic experiences, such as losing a family member, may need to be reconsidered. We instead need to consider how research and PPI endeavours can be designed in non-harmful and appropriate ways to fulfil adolescents’ right to participation.

## Supplementary Material

Supplemental Material

## Data Availability

The data that support the findings of this study are available from the corresponding author, [RR], upon reasonable request.
