# Supplementary material for: Development of a self-management mobile app for bereaved adolescents: evaluating patient and public involvement
Source: Eur J Psychotraumatol. 2024 Jul 12;15(1):2375139. doi: 10.1080/20008066.2024.2375139 (PMC11249156; doi:10.1080/20008066.2024.2375139)
Supplement: Supplemental Material [file ZEPT_A_2375139_SM3328.docx]

**Supplements**

Table 1. Observation schedule

| Theme | Description | Examples of relevant components |
| --- | --- | --- |
| Social context | Take note of the social interactions between teenage research partners and in relation to the researchers, with a particular focus on the power dynamic | 1. Are research partners initiating interaction and discussion with researchers and each other? 2. Are there positive interactions, e.g., humour or appreciation, and negative interactions e.g., invalidation or disregard? 3. How are researchers, research partners and research partners’ input referred to e.g., expert, participant, valuable, helpful? |
| Participation | Take note of the extent and manner of participation of the research partners and of how that participation may change over time | 1. Are research partners participating actively or passively? 2. Are research partners commenting freely or only when asked to? 3. Are there barriers to participation, e.g., difficult language? |
| Influence | Take note of the suggestions made by the research partners and how contributions are approached by the researchers and the group | 1. Are research partners’ contributions relevant and constructive? 2. Are research partners’ suggestions actively considered or overlooked? 3. Are research partners included and active in decision-making? |
